# 75 years of BJAN: honoring our legacy, sharpening the future of anesthesiology research

**DOI:** 10.1016/j.bjane.2026.844773

**Published:** 2026-06-12

**Authors:** Liana Maria Tôrres de Araújo Azi, André Prato Schmidt, Maria José Carvalho Carmona, Maria Angela Tardelli, Mario José da Conceição, Judymara Lauzi Gozzani, Luiz Marciano Cangiani

**Affiliations:** a Universidade Federal da Bahia (UFBA), Hospital Universitário Professor Edgard Santos, Salvador, BA, Brazil; b Universidade Federal do Rio Grande do Sul (UFRGS), Hospital de Clínicas de Porto Alegre, Porto Alegre, RS, Brazil; c Faculdade de Medicina da Universidade de São Paulo (FMUSP), São Paulo, SP, Brazil; d Universidade Federal de São Paulo (UNIFESP), São Paulo, SP, Brazil; e Fundação Universidade Regional de Blumenau, Blumenau, SC, Brazil; f Hospital da Fundação Centro Médico Campinas, Campinas, SP, Brazil

Seventy-five years ago, the first pages of what is now known as the Brazilian Journal of Anesthesiology (BJAN) were printed, driven by the visionary spirit of Renato Correa Ribeiro and the founding members of the Brazilian Society of Anesthesiology (SBA). As Prof. Luiz Marciano Cangiani beautifully chronicles in this issue,^1^ our journal has evolved from a modest national publication into an internationally recognized scientific platform. He reminds us that BJAN’s welcoming pages have always sought to satisfy the “studious curiosity” of our community. Today, as we celebrate this diamond jubilee in issue 76-3, we look back on those years with immense pride and look forward to the future of scientific publishing with renewed enthusiasm.

Over these decades, BJAN has been an important platform for research that has influenced anesthetic practice, accompanying the evolution of our specialty from the era of ether and rudimentary monitoring to the age of precision medicine, ultrasound-guided regional anesthesia, and advanced perioperative monitoring and care. During the past 75 years, our pages have hosted landmark articles that have shaped clinical guidelines and improved patient safety worldwide.^2^^,^^3^

Between 1995 and 2003, several goals were achieved, namely: rigorous bimonthly periodicity; encouragement of national authors; inclusion of courses on scientific methodology at Brazilian Congresses of Anesthesiology; approval of the proposal for the elaboration of structured abstracts submitted at the editors’ meeting held during the world congress in Sydney (1996); indexing in SciELO (Scientific Electronic Library Online); bilingual publication in Portuguese and English (2001); and gradually preparing the journal, following the guidelines of the USA National Library of Medicine, in order to apply for its indexing in that institution.

A truly important step in the global exposure of BJAN was its indexing in the U.S. National Library of Medicine (MEDLINE/PubMed) platform, which was accomplished in 2008. At that time, BJAN had to adapt to the standards of the system, a process that received strong support from SBA Directors and technical assistance from Elsevier, hired in 2006 for this purpose.

After indexing, the large-scale exposure was reflected in the growth of the bibliometric impact. In the past years, BJAN has experienced a consistent improvement in its Impact Factor. Today, BJAN is the top-ranked anesthesiology journal in Latin America, placed in the second quartile (Q2) of the Anesthesiology and Pain Medicine category, with a SCImago Journal Rank (SJR) of 0.537.^4^ This progress reflects the increasing quality of the research published in BJAN since its inception.

The very first article published in BJAN, “Progress in Anesthesia in the Western Hemisphere”, by Ralph M. Waters, was visionary. The manuscript framed anesthesia not merely as a technical act, as it was viewed at the time, but as a medical specialty grounded in physiology, pharmacology, research, and ethical responsibility. Waters also emphasized that anesthesiologists must be competent physicians, fully committed to patient safety and to principles that, more than seven decades later, remain extremely relevant: scientific rigor, professional identity, education, multidisciplinary collaboration, and responsible innovation. These principles continue to define the very best of modern anesthesiology.^5^

Collaborative consensus statements, such as the Update on Perioperative Hypersensitivity Reactions,^6^ the Brazilian Society of Anesthesiology’s Recommendations for Difficult Airway Management in Adults and children,^7^^,^^8^ and the Consensus on Perioperative Transesophageal Echocardiography developed in partnership with the Brazilian Society of Cardiology,^9^ have become highly cited references that actively guide anesthesiologists’ daily practice worldwide. These manuscripts clearly illustrate BJAN’s historical commitment to publishing not only original research but also practical guidelines that bridge the gap between cutting-edge scientific evidence and real-world clinical care, directly translating robust data into safer, more standardized patient outcomes at the bedside.

As we celebrate our history, we are also looking ahead toward the next chapter. Scientific publishing continues to evolve rapidly, demanding greater agility, transparency, and openness. In response, BJAN has launched a targeted Call for Papers to encourage submissions in strategic areas of relevance to our anesthesiology community. Prime examples include our recent Special Issues on Perioperative Care^10^ and Patient Blood Management,^11^ which brought together leading experts to address important contemporary topics in anesthesiology.

During Prof. Maria Carmona’s tenure as Editor-in-Chief, the transition to an English-only format marked a bold and transformative step that firmly established BJAN on the global stage.^12^ This strategic decision has been reflected in the substantial growth to more than 900 submissions annually. This journey continues to gain even greater momentum as we fully embrace the principles of Open Science^13^ and responsibly explore the potential of Artificial Intelligence to enhance our peer-review processes,^14^ all while upholding the rigorous ethical standards that have always defined us.

We are also very proud of our Diamond Open Access model, which is fully funded by SBA. As scientific publishing is becoming increasingly commercialized, BJAN demonstrates that high-quality rigorous research can and should remain freely accessible to readers and authors worldwide, with no financial barriers whatsoever. This model supports broader and equitable access to scientific publishing.

BJAN’s success belongs to all of us. Reaching three-quarters of a century of uninterrupted publication is an impressive milestone that reflects the collective dedication of our Editorial Board, the rigorous expertise of our reviewers, and, above all, the sustained trust and outstanding contributions of our authors. As previously mentioned, our pages have always been open.

Happy anniversary, BJAN!

## Conflicts of interest

The authors declare no conflicts of interest.
